# Modified Universiti Kebangsaan Malaysia Internal Bracing Graft Preparation Technique Using Allograft for Anterior Cruciate Ligament Reconstruction Surgery

**DOI:** 10.1016/j.eats.2025.103982

**Published:** 2025-11-05

**Authors:** Badrul Akmal Hisham Md Yusoff, Mohamed Razzan Rameez, Muhammad Karbela Reza Ramlan, Aliff Omar, Mohamad Azwan Aziz

**Affiliations:** Department of Orthopedic and Traumatology, Faculty of Medicine, Universiti Kebangsaan Malaysia, Kuala Lumpur, Malaysia

## Abstract

Anterior cruciate ligament reconstruction using allografts is limited by delayed biologic incorporation and graft elongation, increasing failure risks during early rehabilitation. To address this, we describe the modified Universiti Kebangsaan Malaysia internal bracing technique, which integrates ultra-high-molecular-weight polyethylene suture tape within the graft construct. This technique aims to provide immediate biomechanical reinforcement, reduce tunnel widening, and accelerate recovery, particularly critical for allografts, which lack the initial strength of autografts. The tendons are then quadrupled over tri-fold loops of FiberTape (Arthrex), which is centrally positioned and secured with FiberWire (Arthrex). The construct is whipstitched with 2.0 Ethibond (Ethicon)—while suture contact with the FiberTape is carefully avoided—and preloaded with an ACL TightRope II Implant (Arthrex) and TightRope Attachable Button System (Arthrex) for femoral and tibial fixation, respectively. Additional GraftLink loop graft construct (Arthrex) stitches are placed at both ends to enhance graft-tunnel integration. This technique uniquely embeds the FiberTape internally within the graft structure to provide biomechanical reinforcement while preventing tunnel abrasion and stress-shielding effects.

Anterior cruciate ligament (ACL) tears are among the most common sports-related injuries. The current standard of care for an ACL rupture is ACL reconstruction. Different types of grafts have been used for ACL reconstruction previously, with autograft and allograft being the mainstays. However, most concerning to orthopaedic surgeons is failure of the ACL reconstruction, due to not only retear but also graft elongation and tunnel widening.

Surgeons have been using different approaches to obtain more stable and biomechanically ideal grafts for ACL reconstruction. Recently, there has been a high degree of interest in applying internal bracing techniques for ACL reconstruction, with good clinical outcomes. Different types of internal brace augmentation of ACL reconstruction are performed by combining ACL graft with fiber tape, which has been shown to improve the biomechanical properties but not the overall strength of the graft.^1^ We present our surgical technique for preparation of the modified Universiti Kebangsaan Malaysia (UKM) internal bracing graft, which can yield better outcomes after ACL reconstruction.

## Surgical Technique

### Patient Positioning

The patient undergoes spinal anesthesia and is positioned supine with the leg hanging down from the knee joint, which allows 120° of flexion of the knee. Standard portals are created for diagnostic arthroscopy of the knee to visualize the torn ACL graft and any associated injury to the knee joint.

### Graft Preparation

**Fig 1:**
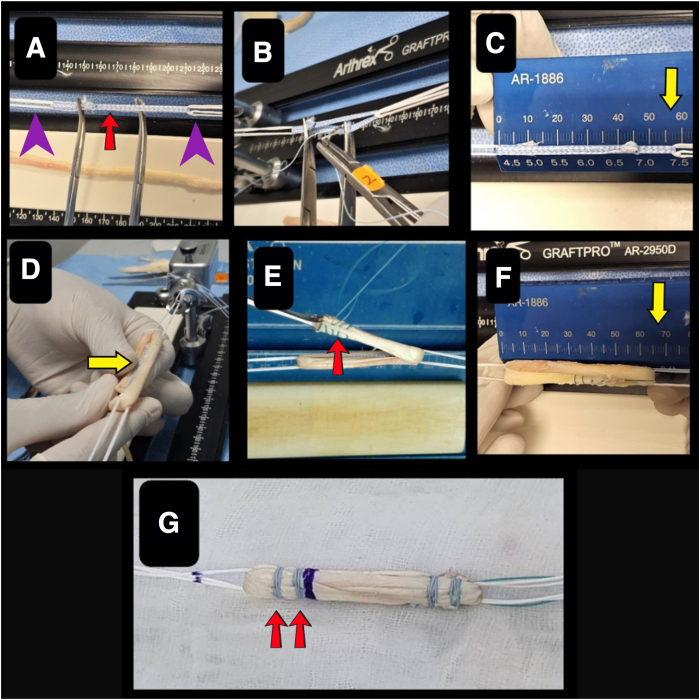
Steps for graft preparation. (A) Loading of ACL TightRope implants onto GraftPro base and attachments. The ACL TightRope II RT and ACL TightRope Attachable Button System (purple arrowheads) are looped 1.5 times around the GraftPro base. Then, a tri-fold loop of FiberTape (red arrow) is placed on the GraftPro. (B) The FiberTape is secured with a No. 0 FiberWire. (C) The final trimmed length of the FiberTape measures 58 mm (yellow arrow). (D) The graft is folded in a quadruple configuration for optimal load distribution, embedding the FiberTape internally (yellow arrow). (E) Both ends of the graft are whipstitched together using 2.0 Ethibond sutures (red arrow). (F) The final graft length is adjusted to 65 mm (yellow arrow). (G) The GraftLink loop construct technique is applied, with 2 reinforcing sutures (red arrows) placed on either side of the graft for additional stability.

Graft preparation and tensioning are conducted using a specialized graft preparation station (GraftPro [AR 2950D]; Arthrex), as shown in Figure 1. The Achilles tendon allograft is favored for its superior dimensional properties compared with other allograft alternatives. For patients with an average height of 155 to 179 cm, the graft length should measure at least 65 mm. In smaller patients (height <155 cm), a smaller graft length of 62 mm is recommended, with an internal bracing length of 62 mm. For taller patients (height >179 cm), a longer graft length of 67 mm is recommended, with an internal bracing length of 60 mm.

The ACL TightRope implants are loaded onto the graft preparation station (GraftPro), with a TightRope implant with a flipping suture (ACL TightRope II implant [AR1588RT-2J]; Arthrex) designated for femoral fixation and an ACL TightRope Attachable Button System (ABS; Arthrex) for tibial fixation. Next, tri-fold loops of 2-mm ultra-high-molecular-weight polyethylene fiber tape (FiberTape; Arthrex) are secured to the graft preparation station (GraftPro) using No. 0 FiberWire (Arthrex), ensuring that the FiberTape remains free from the ACL TightRope implants to preserve their gliding functionality. After fixation, the FiberTape is trimmed to its final length. The graft is then symmetrically folded in a quadruple configuration over the FiberTape loops, embedding the tape internally, and the ends are whipstitched with 2.0 Ethibond suture (Ethicon). Care is taken to avoid capturing the FiberTape or TightRope implants during suturing. The whipstitch is tensioned by passing the suture ends alternately over and under the graft loops, compacting the graft to the target, ranging from 62 to 67 mm in length, before final fixation to the preparation station.

To optimize graft integration, stump-end stitches are critical for securing both the graft tissue and the modified UKM internal bracing construct while maximizing the surface area for Sharpey fiber formation within the sockets. By use of the ACL GraftLink loop construct technique from Arthrex,^2^ two additional stitches are placed on each side of the graft to reinforce stability. Table 1 describes the steps for graft preparation. The graft is marked at predetermined locations corresponding to the required intraosseous lengths for both femoral and tibial tunnel fixation. Graft preparation is also highlighted in Video 1.

**Table 1:** Steps for Graft Preparation

1. The Length of the graft should be at least 65 mm in total for an average height of 155-179 cm.
2. The ACL TightRope implants are loaded onto the GraftPro base and attachments, with a TightRope II RT implant for femoral fixation and an ACL TightRope ABS implant for tibial fixation.
3. Tri-fold FiberTape is loaded onto the GraftPro base and sutured using No. 0 FiberWire. At this point, the individual preparing the graft must avoid suturing the FiberTape to the TightRope implants or “catching” the FiberTape; doing so will not allow shortening of the TightRope because it will not be able to glide through the implants.
4. After both ends of the FiberTape are secured using FiberWire, the final length should be 58 mm.
5. The harvested graft is prepared after removing the remaining muscle and fat.
6. The graft is loaded through the implants by folding in a quadruple configuration over the loops such that the FiberTape will be incorporated inside the graft.
7. Both ends are stitched together with 2.0 Ethibond suture using a whipstitch technique. Suturing the FiberTape or the TightRope implants should be avoided while suturing both ends.
8. The 2 ends of the whipstitch should go over and under the graft loop for tensioning and to tuck the graft inside.
9. Once the graft is folded and the length is 65 mm, the whipstitch suture is secured.
10. Stump-end stitches are important to ensure the maximum hold of both the graft tissues and the MUIB, as well as to maximize the surface area for Sharpey fiber integration to the graft stump within both sockets. Via the GraftLink technique, 2 stitches are placed on either side of the graft.

ABS, Attachable Button System; MUIB, modified UKM internal bracing; UKM, Universiti Kebangsaan Malaysia; ACL, Anterior Cruciate Ligament

### All-Inside ACL Reconstruction Technique

The all-inside ACL reconstruction procedure follows the established method of Lubowitz et al.^3^ The graft is initially introduced into the femoral socket and secured using a TightRope RT suspensory button device (ACL TightRope II Implant). After confirmation that the button has traversed the predetermined femoral intraosseous distance as demarcated by a methylene blue marking on the loop, the device is deployed on the lateral femoral cortex. Sequential tensioning of the shortening sutures in alternating fashion facilitates optimal graft seating within the femoral socket.

For tibial fixation, the graft terminus incorporating the ABS button construct, along with associated graft sutures and the free ends of the FiberTape internal brace, is delivered into the tibial tunnel via passing suture. With the knee positioned in 0° of extension through manual foot stabilization, the TightRope shortening sutures are secured to the ABS button to achieve primary fixation. In patients with hyperlaxity, the knee will be positioned at negative zero degree extension through manual foot stabilization.

The construct undergoes several cycles of knee motion to ensure proper tensioning, followed by final tightening of both the femoral and tibial TightRope shortening sutures with the knee maintained in full hyperextension. Supplemental fixation is achieved by tying the TightRope shortening sutures and both ends of the fiber loop sutures to the ABS button.

### Postoperative Rehabilitation

The postoperative rehabilitation protocol follows a phased, evidence-based approach with immediate intervention to address arthrogenic muscle inhibition. Beginning on postoperative day 1, we implement targeted strategies to minimize arthrogenic muscle inhibition, including multimodal analgesia for pain control, cryotherapy and compression to reduce swelling, and carefully graded range-of-motion exercises within protected limits. Patients maintain protected weight-bearing for the first 6 weeks to safeguard graft integrity while promoting early neuromuscular activation.

After this initial phase, rehabilitation progresses systematically. Range of motion is restored gradually, with particular emphasis on achieving full extension early while cautiously increasing flexion. Weight-bearing is advanced as tolerated, contingent on demonstration of adequate quadriceps function and lower-extremity control. Closed-chain strengthening exercises are prioritized to enhance joint stability and proprioception while minimizing shear forces across the healing graft.

## Discussion

**Fig 2:**
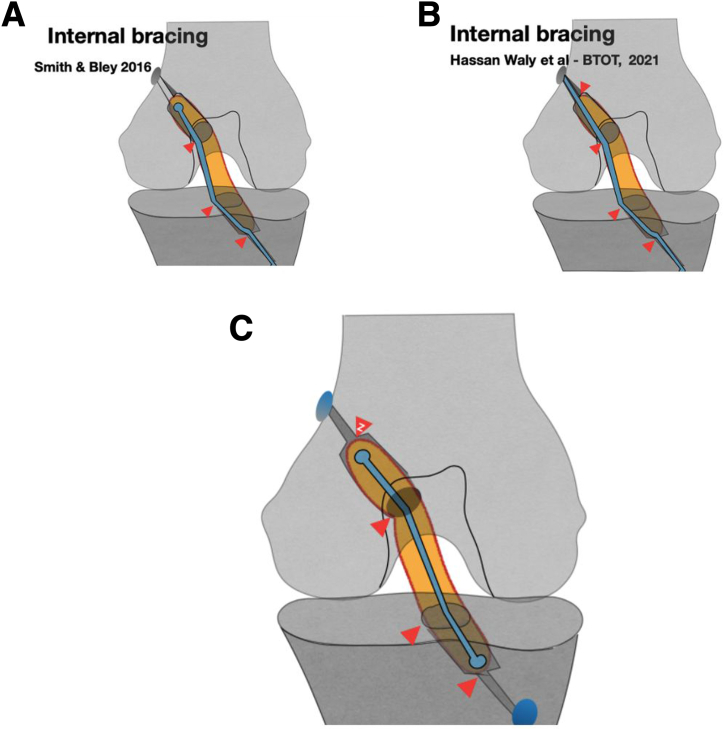
Comparison between the three technique of anterior cruciate ligament (ACL) internal bracing. (A) Internal bracing technique of Smith and Bley.^4^ The internal brace is fixated separately from the graft to reduce the risk of overconstraining the joint. However, this creates 3 points of contact pressure, thus increasing the risk of tunnel widening and graft elongation through the bungee and windshield-wiper effects. (B) Internal bracing technique of Waly et al.,^5^ modified by using a crossing configuration. This method further increases the points of contact pressure to 4. (BTOT, button tie-over technique.) (C) Modified Universiti Kebangsaan Malaysia internal bracing technique, in which the internal brace is placed at the center and the contact pressure point is eliminated.

In 2016, Smith and Bley described ^4^ an allograft preparation technique that incorporates a collagen-coated, ultra-high-molecular-weight polyethylene–polyester composite suture tape as an internal brace to augment ACL reconstruction, performed via the all-inside technique. They outlined that the internal brace is fixated separately from the graft to reduce the risk of overconstraining the joint. However, this creates points of contact pressure, increasing the risk of tunnel widening and graft elongation through the bungee and windshield-wiper effects, as shown in Figure 2A. Waly et al.^5^ modified the internal bracing technique by performing a crossed configuration. This configuration results in additional contact pressure and a further increase in graft failure (Fig 2B). On the basis of our understanding of the contact point, we have modified the technique by placing the internal bracing at the center and eliminating the contact pressure point (Fig 2C). This type of graft preparation offers an advantage over the other internal bracing techniques because the FiberTape used for internal bracing is incorporated within the graft and, hence, tunnel widening is reduced. We postulate, using a simulated model, that the eccentrically placed internal bracing outside the graft will subject the sockets to abrasion during the forceful and repetitive flexion-extension motion of the knee, as opposed to a centrally placed internal brace. The FiberTape is incorporated within the graft to prevent it from touching the tunnel wall during knee motion. Excessive movement of the FiberTape may widen the socket in the femur or tibia because it has a smaller size in relation to the socket. Graft elongation through the bungee effect (up and down) and windshield-wiper effect (side to side) has increased the rate of post-ACL reconstruction failure. The aforementioned advantages are highlighted in Table 2. The implementation of this technique provides superior biomechanical reinforcement, which reduces the incidence of tunnel widening and permits an accelerated postoperative rehabilitation protocol (Table 3).

**Table 2:** Advantages and Disadvantages of MUIB Allograft Preparation

Advantages
Eliminates contact pressure, reducing bungee and windshield-wiper effects
Provides constant graft length
Offers similar or better strength compared with autograft
Provides shorter duration of surgery
Reduces harvesting procedure complications
Eliminates pain from harvesting procedure
Allows earlier return to play
Disadvantages
Slightly higher risk of infection
Potential stress shielding (which can potentially be reduced through graft preparation with quadruple configuration)
Higher cost of operation

MUIB, modified UKM internal bracing; UKM, Universiti Kebangsaan Malaysia.

**Table 3:** Pearls and Pitfall of MUIB Allograft Preparation

Pearls
Biomechanical reinforcement: The internal placement of FiberTape within the graft provides immediate load sharing, reducing early graft elongation and protecting the allograft during the vulnerable incorporation phase.
Reduced tunnel widening: By embedding the FiberTape centrally, abrasion against the tunnel walls is minimized, potentially lowering the risk of tunnel expansion.
Simplified graft preparation: Standardized measurements and clear steps enhance reproducibility.
Early rehabilitation feasibility: The construct's stability may allow accelerated postoperative rehabilitation protocols without compromising graft integrity.
Pitfalls
Technical precision required: Misplacement of FiberTape or accidental suturing to TightRope implants could impair implant function or graft mechanics.
Learning curve: Surgeons unfamiliar with internal bracing may require training to avoid errors in tape looping, suturing, or tensioning.
Cost implications: Use of multiple high-end implants may increase procedural costs compared with conventional techniques.

MUIB, modified UKM internal bracing; UKM, Universiti Kebangsaan Malaysia.

The described graft technique yields potentially superior outcomes in minimizing tunnel widening while maintaining comparable strength to autograft. In addition, this approach may decrease operative time and mitigate complications associated with graft harvesting, including the risk of permanent muscular strength deficits.

## Disclosures

The authors declare the following financial interests/personal relationships which may be considered as potential competing interests: B.A.H.M.Y. has a patent pending (UKM.IKB.800-4/1/4815). All other authors (M.R.R., M.K.R.R., A.O., M.A.A.) declare that they have no known competing financial interests or personal relationships that could have appeared to influence the work reported in this paper.

## Supplementary Data

Video 1 Sequential steps for preparing modified UKM internal bracing (MUIB) allograft. First, the ACL TightRope implants are loaded onto the GraftPro base and its attachments. Next, an ACL TightRope II RT and ACL TightRope Attachable Button System are carefully looped approximately 1.5 times around the base of the GraftPro. Then, FiberTape is attached to the GraftPro in a tri-fold loop and is subsequently secured firmly into place using a No. 0 FiberWire. The graft itself is folded in a quadruple configuration to ensure optimal load distribution across the construct. Then, both ends of the graft are meticulously whipstitched together using a strong No. 2-0 Ethibond suture. The final prepared graft is adjusted to achieve a total length of 65 mm. To complete the process, the GraftLink technique is performed, which involves placing 2 separate reinforcing sutures on either side of the graft to provide enhanced stability. This technique embeds the internal bracing inside the graft, reducing the contact pressure inside the tunnel.
